# Bovine Alphaherpesvirus 1, Bovine Alphaherpesvirus 5 and Bubaline Alphaherpesvirus 1 in Palatine Tonsils from Water Buffaloes in Northern Brazil and Possible Links with the Origin of Bovine Alphaherpesvirus Type 5

**DOI:** 10.3390/v16071024

**Published:** 2024-06-26

**Authors:** Bruna Paredes-Galarza, Martha T. Oliveira, Francine B. Timm, Nicole V. Stone, Lina Violet-Lozano, Richard S. Salvato, Nícolas D. Müller, Bruno A. Prandi, Raíssa Gasparetto, Michelen Gonçalves, María A. S. Teixeira, Márcio A. O. Moura, Gabriela Riet-Correa, Valíria D. Cerqueira, Pedro S. Bezerra, Fabrício S. Campos, Ana C. Franco, Paulo M. Roehe

**Affiliations:** 1 Laboratório de Virologia, Departamento de Microbiologia, Imunologia e Parasitologia, Instituto de Ciências Básicas da Saúde, Universidade Federal do Rio Grande do Sul, Rua Ramiro Barcelos, 2600, Porto Alegre CEP 90035-003, RS, Brazil; brunaparedes97@gmail.com (B.P.-G.); nicolevcvc@hotmail.com (N.V.S.);; 2 Centro de Desenvolvimento Científico e Tecnológico (CDCT), Centro Estadual de Vigilância em Saúde (CEVS) da Secretaria Estadual da Saúde do Rio Grande do Sul (SESRS), Porto Alegre CEP 90450-190, RS, Brazil; 3 Laboratório de Virologia, Instituto de Pesquisas Veterinárias Desidério Finamor, Secretaria da Agricultura, Pecuária e Irrigação, Estrada do Conde, 6000, Eldorado do Sul CEP 92990-000, RS, Brazil; 4 Agência Estadual de Defesa Agropecuária do Estado do Pará (ADEPARÁ), Belém CEP 66080-008, PA, Brazil; 5 Laboratório de Patologia Animal, Instituto de Medicina Veterinária, Universidade Federal do Pará (UFPA), Castanhal CEP 68740-970, PA, Brazil

**Keywords:** water buffaloes, bovine herpesvirus, cattle

## Abstract

Herpesviruses are significant pathogens of ruminants. In water buffaloes (*Bubalus bubalis*), however, herpesviruses have not been thoroughly studied. Although bubaline alphaherpesvirus 1 (BuAHV1) and bovine alphaherpesvirus 1 (BoAHV1) have already been recovered from water buffaloes, to date, no reports on the occurrence of bovine alphaherpesvirus 5 (BoAHV5) in these animals have been published. Therefore, the aim of this study was to search for BuAHV1, BoAHV1, and BoAHV5 in palatine tonsils of apparently healthy water buffaloes from the Pará state, Northern Brazil. Tissue samples of tonsils (*n* = 293) were screened by a nested PCR (nPCR) targeting a region of *UL44* (gC coding gene), followed by sequencing, to detect and differentiate between the viral types. Viral genome segments were detected in 18 out of 293 (6.1%) of the palatine tonsil samples. Two animals carried genomes of BoAHV1 only, eleven animals carried BoAHV5 genomes only, and four animals carried BuAHV1 only. Another animal had both BoAHV1 and BoAHV5 genomes in its tonsils. No infectious virus could be recovered from any of the samples. The BuAHV1 sequences identified here were more closely related to BuAHV1 genomes identified in India. Phylogenetic analyses suggested a closer relationship between the recovered BoAHV5 and BuAHV1 genomes. Therefore, evidence is provided here to confirm that not only BoAHV1 and BuAHV1, but also BoAHV5, can infect water buffaloes. This report highlights (i) the first detection of BoAHV5 in water buffaloes and (ii) the occurrence of coinfections with BoAHV1 and BoAHV5 in that species. Such findings and the similarity of BoAHV5 to Indian herpesvirus genomes suggest that the origin of type 5 may be linked to recombinations between bovine and bubaline herpesviruses within bubalines, since the scenario for generation of recombinants in buffaloes is potentially present.

## 1. Introduction

Alphaherpesviruses are important pathogens of ruminants, capable of causing significant productive and economic losses. Bovine alphaherpesvirus 1 (*Varicellovirus bovinealpha1*, BoAHV1), bovine alphaherpesvirus 5 (*Varicellovirus bovinealpha5*, BoAHV5), and bubaline alphaherpesvirus 1 (*Varicellovirus bubalinealpha1*, BuAHV1) are classified within the order *Herpesvirales*, family *Orthoherspesviridae*, subfamily *Alphaherpesvirinae*, genus *Varicellovirus* [1]. A key characteristic of all alphaherpesviruses is their ability to establish lifelong latency in various cell types, such as neurons, lymphocytes, trigeminal ganglia, and palatine tonsils. Alphaherpesviruses are responsible for a variety of conditions, including reproductive, respiratory, and neurological diseases [2,3]. Usually, such viruses cause mild or asymptomatic infections in their original hosts, where occasional clinical signs are the visible tip of the iceberg. Under stressful situations, infections can be reactivated, eventually with induction of disease, leading to virus shedding through secretions and excretions [4,5,6,7,8]. Such a mechanism is a remarkable example of herpesvirus evolution, which leads to minimal disease and facilitates transmission of the virus to other susceptible hosts, thus ensuring the perpetuation of the infection.

The most recognized alphaherpesvirus in cattle is BoAHV1; this virus, first recognized in the sixties [9], is distributed worldwide, being a major cause of disease in cattle [10,11,12]. BoAHV1 infection can result in the classical condition known as infectious bovine rhinotracheitis (IBR), a respiratory disease primarily characterized by superior respiratory tract infection, tracheitis, conjunctivitis, fever, dyspnea, coughing, nasal discharge, enteritis, and, occasionally, encephalitis [13,14]. In addition, the virus is manifested in bulls as infectious pustular balanoposthitis (IPB) and in heifers as infectious pustular vulvovaginitis (IPV), resembling genital disease associated with herpes simplex virus type 1 (HSV1) infections in humans [15]. In addition, a great deal of losses are associated with reproduction, which can be manifested as embryo losses and abortions, on occasions leading to “abortion storms”, often as a result of semen contamination, where the virus can be transmitted either by natural mating, artificial insemination, or embryo transfers [16,17,18,19].

In water buffaloes (*Bubalus bubalis*), BoAHV1 has been shown to be associated with subclinical disease [19,20]. However, Fusco and colleagues identified BoAHV1 genomes in 11 aborted buffaloes, in the tissues of a calf born with hind limb deformity, and in the tissues of two stillborn calves. The authors suggested that BoAHV1 was responsible for these clinical outcomes [21].

In bovine (*Bos taurus taurus*) and zebuine (*Bos taurus indicus*) species, BoAHV5 also induces mostly inapparent infections. When clinical disease ensues, it primarily causes a usually fatal necrotizing meningoencephalitis in bovine cattle. The disease is characterized by clinical signs such as apathy, anorexia, bruxism, ptyalism, circling, head pressing against objects, tongue protrusion, blindness, seizures, and recumbency. In younger calves, fatality is usually high; this can reach up to 100% [22,23,24]. Occasionally, BoAHV5 has been shown to be associated with reproductive disorders in affected heifers [25,26,27]. To date, BoAHV5 has never been reported to infect water buffaloes. 

The natural host of BuAHV1 is the water buffalo. BuAHV1 typically causes asymptomatic infections in buffaloes and mild acute respiratory signs [28,29,30,31]; however, Amoroso et al. (2013) reported the detection of BuAHV1 DNA in an aborted buffalo in Italy [32]. 

Water buffalo raising is gaining popularity worldwide as alternative livestock production. Currently, Brazil has the largest population of buffaloes in the Americas, with about 1,598,268 heads [33]. In the current scenario of livestock farming in Brazil and other countries, buffalo and cattle are sometimes co-housed and/or raised together or in proximity. This creates opportunities for infectious agents to cross-infect or spillover to other hosts. This is particularly concerning for herpesviruses, as some ruminant alphaherpesviruses are known to, occasionally, cross the species barrier [34]. Currently, information on the epidemiological role of bovine or bubaline herpesvirus cross-infections in bovine, zebuine, or bubaline species is limited. In view of such a gap, the present work was conducted to investigate the occurrence of BoAHV1, BoAHV5, and BuAHV1 in Brazilian water buffaloes by examining their palatine tonsils.

## 2. Materials and Methods

### 2.1. Tissue Sampling 

**Figure 1:**
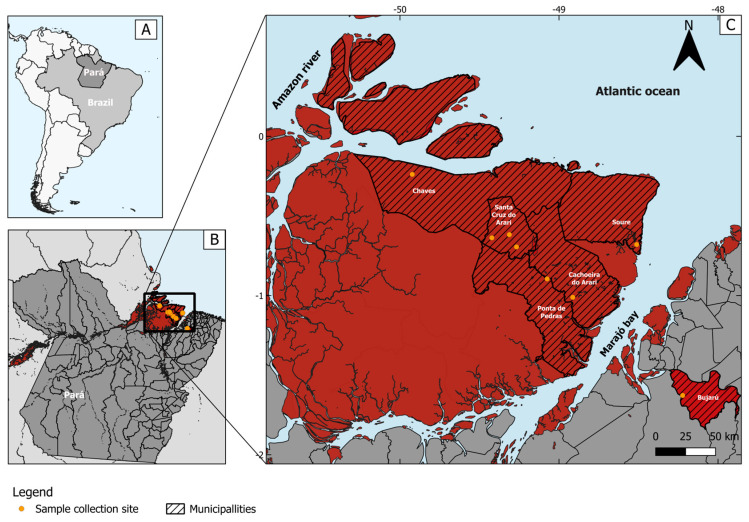
Locations of origin of herds from which samples were collected. (**A**) Map of Brazil (light gray), highlighting Pará state (dark gray). (**B**) Map showing Pará state (dark gray), highlighting Marajó island (in red). (**C**) Map of part of the Marajó archipelago, focusing on the main island, and highlighting municipalities of origin of the sampled animals (in red with black stripes); the yellow dots represent the location of the farms of origin of the sampled animals.

The samples used for this investigation were originally collected in 2020. Palatine tonsil samples were taken from clinically healthy water buffaloes (*Bubalus bubalis*) in an abattoir. Samples (*n* = 243) were taken from animals from 8 different herds from 6 municipalities in the Marajó archipelago, at the delta of the Amazon River, an area of approximately 40.100 km^2^, divided in 16 municipalities in the state of Pará, Northern Brazil. In addition, 50 tonsil samples (*n* = 50) were collected from buffaloes from a continental municipality (Bujaru, Pará) (Figure 1). The tonsils were collected individually and stored in sterile tubes under refrigeration throughout the collection process until transportation to the laboratory; the storage duration did not exceed 6 h under these conditions. Once at the laboratory, the samples were initially stored at −20 °C, remaining at this temperature for nearly 22 months, and then transferred to −80 °C.

### 2.2. DNA Extractions 

The DNA extraction from 293 palatine tonsils was performed as previously reported [35,36], but with small modifications as follows. Briefly, 40 mg of the tissue sample was minced with scalpel blades and mixed in lysis buffer [(20 mM Tris-HCl, 10 mM EDTA, 200 mM NaCl); 100 ug Proteinase K; 10% SDS (sodium dodecyl sulfate)]. This mix was incubated overnight at 56 °C. The homogenates were clarified by centrifugation at 12.000× *g* for 10 min and 400 µL of the supernatant were collected. DNA was extracted using one volume of buffer-saturated phenol (Invitrogen, Waltham, MA, USA) and 40 µL of 5 M NaCl followed by incubation at room temperature for 30 min. After centrifugation at 12.000× *g* for 10 min, the supernatant was transferred to a fresh tube and 2.5 volumes of 100% ethanol was added. Next, samples were incubated at −20 °C for 30 min. Finally, the DNA was eluted in 200 µL of TE (10 mM Tris-HCl; 1 mM EDTA) and stored at −20 °C until further use.

### 2.3. PCR for Detection of UL44 (Glycoprotein C) Gene

DNA samples were subjected to a nested polymerase chain reaction (nPCR) using two sets of primers (external and internal) for amplification of a region of the *UL44* gene (glycoprotein C) as described by [36]. This reaction targets the three virus types (BoAHV1, BoAHV5 and BuAHV1); however, it does not differentiate between BoAHV5 and BuAHV1. Briefly, a first round of PCR was performed with an external primer set (PF2: 5′—CGGCCACGACGCTGACGA—3′) and (PR1: 5′—CGCCGCCGAGTACTACCC—3′), targeting a fragment of about 570 bp (equivalent of 38.5% gene length) in BoAHV1, BoAHV5 and BuAHV1. Subsequently, a second-round or nested PCR (nPCR) with the products of the first PCR was carried out to differentiate between BoAHV1 and the two other virus types were used in two type-specific reactions. In the first nPCR, an internal primer set specific for BoAHV1 (PF6: 5′—CTAACATGGAGCGCCGCTT—3′ and PR3: 5′—CGGGGCGATGCCGTC—3′) was used. To detect BoAHV5 or BuAHV1, a second nPCR was carried out with an internal primer set for BoAHV5 (PF7: 5′—GTGGAGCGCCGCTTCGC—3′ and PR2: 5′—TATCGCGGAGAGCAGGCG—3′). These reactions are expected to generate amplicons of 161 bp for BoAHV1 and 236 bp for both BoAHV5 and/or BuAHV1. Yet, these do not differentiate BoAHV5 from BuAHV1. To differentiate these two viruses, next-generation sequencing (NGS) of the amplicons obtained was performed.

For all reactions, 1 μL of the sample (either DNA or the product of the first PCR) was used as a template. In all runs, the reactions were made up to a final volume of 25 μL, containing 10× Taq polymerase buffer (Invitrogen), 1 mM MgCl_2_, 0.2 mM of each dNTP, 0.2 µM of each primer, 1 U Taq DNA polymerase (Invitrogen), and 2.5% dimethylsulfoxide (DMSO). For the first-round reaction, the following conditions were used: initial denaturation at 94 °C for 5 min, 35 cycles of first denaturation at 94 °C for 1 min, annealing at 62 °C for 1 min, extension at 72 °C for 1 min, and a final extension step at 72 °C for 5 min. For the second-round (nPCR) reactions, cycling was performed as follows: initial denaturation at 94 °C for 3 min, 35 cycles of first denaturation at 94 °C for 15 s, annealing at 62 °C for 30 s, extension at 72 °C for 30 s, and a final extension step at 72 °C for 5 min. The PCR products were analyzed by electrophoresis on 1.5% agarose gel stained with 1× DSView^TM^ Nucleic Acid Stain (Sinapse Biotecnologia, São Paulo, Brazil). Samples were run at 80 mV for 30 min in 1× TAE buffer along with a 50 pb molecular weight marker (MWM) (Ludwig Biotec, Porto Alegre, Brazil).

### 2.4. Next-Generation Sequencing (NGS)

To confirm the positive samples and to differentiate between BoAHV5 and BuAHV1, all nPCR-positive samples were subjected to NGS. Illumina NGS libraries were prepared from each sample with Illumina COVIDSeq (Illumina INC., EUA) according to the manufacturer’s instructions. Libraries were purified with AMPure XP (Benchman Coulter, EE. UU.) and then quantified with Qubit dsDNAHS kit (Invitrogen, EE. UU.). Samples were sequenced on an Illumina MiSeq instrument with the MiSeq Reagent Kit v3, for a total of 600 cycles (Illumina, San Diego, CA, USA). Bioinformatic analyses of the obtained read files were performed by Map to Reference Alignment (reference sequences used: KU198480 for BoAHV1, KY549446 for BoAHV5 and NC_043054 for BuAHV1) using the Geneious software (v.R9.1.8).

### 2.5. Sequencing and Phylogenetic Analysis

MAFFT alignment was performed using Geneious Software (v.R9.1.8). Phylogenetic relationships were inferred by the maximum-likelihood method, using IQ-TREE v2.0.3 Database [37,38] with the 1000 bootstrap replicates setting. The phylogenetic tree was designed in FigTree v1.4.4. The sequences of other Alphaherpesvirus used in this analysis are available in GenBank. The accession numbers can be found in Table S1.

### 2.6. Cell Culture

Madin-Darby bovine kidney (MDBK) cells were cultured in Dulbecco’s Modified Eagle’s Medium (DMEM) supplemented with 10% fetal calf serum (FCS), 1000 U/mL penicillin, and 50 µg/mL streptomycin. Cells were maintained at 37 °C in a 5% CO_2_ humid atmosphere. One day prior to inoculation, 10^5^ cells per well were seeded in 24-well plates. 

### 2.7. Virus Isolation

For virus isolation attempts, 40 mg of tonsillar tissue samples were minced with scalpel blades, suspended in 1 mL of DMEM, and filtrated through a 0.45 μm pore size filter. Samples were clarified, and 200 µL was used to inoculate 70–80% confluent monolayers of MDBK cells cultures in 24-well plates. Cells were incubated at 37 °C for 1 h for adsorption, followed by the addition of fresh DMEM supplemented with antibiotics (1000 U/mL penicillin and 50 µg/mL streptomycin) and 5% FCS free of herpesviruses antibodies. Mock infected cells were inoculated with 200 µL of fresh DMEM supplemented as above, used as a negative control. Plates were incubated at 37 °C in a 5% CO_2_ atmosphere, and cells were examined daily in an optical microscope and compared with controls in search for cytopathic effects (CPE). After 7 days of incubation, the plates were frozen at −80 °C and thawed once. The supernatant medium was harvested, and 200 µL was inoculated onto a fresh monolayer of MDBK cells. Five blind passages in culture were performed. Samples that did not exhibit CPE up to the fifth blind passage were considered negative for virus isolation.

## 3. Results

### 3.1. Virus Genome Detection 

All palatine tonsil samples were examined in the search for BoAHV1, BoAHV5, and BuAHV1 by nPCR. Viral genome segments were detected in 18 out of 293 (6.1%) of the palatine tonsil samples. Two samples contained BoAHV1 genomes only; eleven samples contained BoAHV5 genomes only, and four samples contained BuAHV1 genomes only. One sample contained genomes of BoAHV1 and BoAHV5. All results were subsequently confirmed by sequencing, including the sample where the co-infection was identified. All sequenced segments were deposited at GenBank (access numbers can be found in Table S1). Out of the 18 positive samples, 1 was from a continental farm and 17 originated from six different farms from the Marajó archipelago.

### 3.2. Virus Isolation 

Virus isolation attempts were performed with all 18 nPCR-positive tonsil samples. No infectious virus could be recovered from the samples by inoculation on MDBK cells. During the five consecutive blind passages, no CPE was observed in the cultured cells.

### 3.3. Analyses of the UL44 Gene (gC)

**Figure 2:**
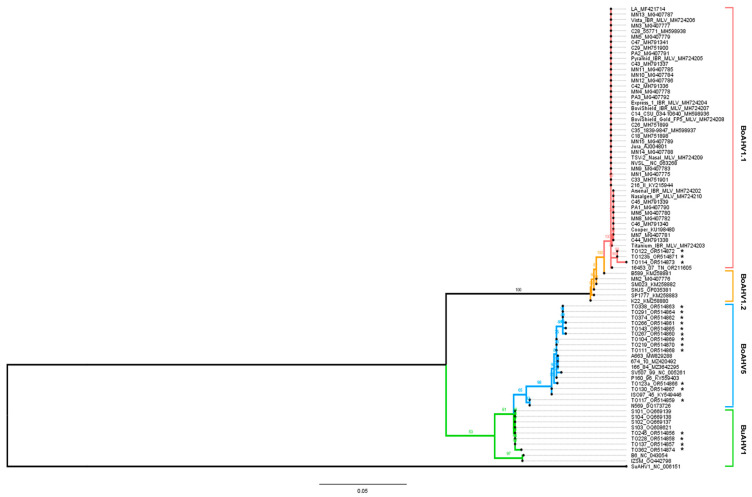
Phylogenetic tree based on analysis of the partial sequences coding for gC (*UL44*). The sequenced region corresponds to 37.13% of the total *gC*-coding gene for BoAHV1 (567 nt), 38.60% for BoAHV5 (564 nt), and 39% for BuAHV1 (564 nt). Each sequence (marked with a black dot) is followed by its name and the GenBank accession number. Red: BoAHV1.1; yellow: BoAHV1.2; blue: BoAHV5; green: BuAHV1. The Suid alphaherpesvirus 1 (SuAHV1) glycoprotein C gene was used as an outgroup. Sequences generated in this study are marked with an asterisk. Phylogenetic analysis was performed using the maximum-likelihood method in IQ-TREE v2.0.3 Database with 1000 bootstrap replicates setting. The phylogenetic analysis also included sequences of the equivalent region of other alphaherpesviruses available at GenBank. * *UL44* sequences of BoAHV1, BoAHV5, and BuAHV1 recovered in this study.

The BoAHV1, BoAHV5, and BuAHV1 complete gC sequences comprise 1527 nucleotides (nt), 1461 nt, and 1443 nt, respectively. The partial region of the gC gene sequenced here corresponds to 37.13% of the complete gene for BoAHV1 (567 nt), 38.60% for BoAHV5 (564 nt), and 39% for BuAHV1 (564 nt). Phylogenetic analysis of sequences obtained in this study reveal that three of the BoAHV1 sequences recovered (samples TO114, TO122, and TO 123b) presented 99.4 to 100% identity among themselves. Moreover, these clustered along with the BoAHV1.1 reference strain Cooper (NC_063268), with which these displayed 99.1 to 99.6% identity (Figure 2). Therefore, TO 123b, TO 122, and TO 114 were confirmed as BoAHV1 genomes.

The 12 BoAHV5 sequences obtained in this study clustered into two different groups. Eleven BoAHV5 sequences (samples TO 104, TO 111, TO 123a, TO 130, TO143, TO 219, TO266, TO 267, TO 291, TO 338, and TO 374) presented 99.1% to 99.8% identity among themselves and clustered together with the South American BoAHV5 strains (A663, 166/84, 674/10, P160/96, ISO 97/45), presenting 98.7% to 99.2% identity with the SV507/99 Brazilian reference strain (NC_005261). The other BoAHV5 sequence (sample: TO 117) clustered with an Australian BoAHV5 strain (N569) with 98% identity (Figure 2).

One sample was positive for both BoAHV1 and BoAHV5. The BoAHV1 sequence (sample TO 123 b) clustered together with the BoAHV1.1 reference strain Cooper (NC_063268). The BoAHV5 sequence (sample TO 123a) was clustered together with the South American BoAHV5 sequences.

Finally, the four BuAHV1 sequences recovered here (TO 245, TO 228, TO 362, and TO 137) presented 99.6% to 100% identity among themselves. Phylogenetic analyses of equivalent sequences available at GenBank reveal that that BuAHV1 isolates could be separated into two different clusters: one of these included the four reported sequences of Indian origin, and another comprised sequences recovered from buffaloes from Italy (IZSM, OQ442798) and Australia (B6, NC_043054). Interestingly, the sequences recovered in the present study clustered with Indian BuAHV1 strains S101, S102, S103, and S104, with high identity [99.6% (TO 362) to 100% (TO 137, TO 228, and TO 245)]. In addition, all BuAHV1 sequences obtained here were more closely related to BoAHV5 (95.3% to 95.7% identity) than to BoAHV1 (91.3% to 91.7% identity; Figure 2, Table S3).

## 4. Discussion

In this study, a search for BoAHV1, BoAHV5, and BuAHV1 genomes in palatine tonsils of water buffaloes was conducted. Genome segments were detected in 18 out of 293 (6.1%) clinically healthy water buffaloes from different farms in Northern Brazil. Eleven (3.75% buffaloes were found to carry BoAHV5 DNA; four (1.36%) animals contained BuAHV1 DNA; two (0.68%) contained BoAHV1 DNA, and one (0.34%) animal was co-infected with BoAHV1 and BoAHV5. Although other authors have reported BoAHV1 infections in sheep, goats, cervids, and buffaloes [39,40,41], this is the first report on BoAHV5 detection in water buffaloes, confirming the susceptibility of water buffaloes not only to BoAHV1 and BuAHV1, but also to BoAHV5 [19,20,21]. It is interesting to compare our results with those similar studies conducted in trigeminal ganglia of cattle from Brazil and Uruguay. In Brazil, in that study [31], bovine herpesvirus genomes were detected in 87% of the sampled animals, of which 82.8% had BoAHV1, 93.1% had BoAHV-5 and 75.9% had both BoAHV1 and BoAHV5 genomes, suggesting a higher prevalence of BoAHV5 than BoAHV1. In Uruguay, 64.5% of the sampled cattle contained herpesvirus genomes, of which 48.4% had BoAHV1, 1.6% had BoAHV5, and 14.5% had co-infections with BoAHV1 and BoAHV5 [42]. The low prevalence of BoAHV1, BoAHV5, and BuAHV1 in tonsils suggested that, although tonsils may have served as site of infection or latency for herpesviruses, such tissues are not the primary site of latency, as was previously observed [31,42]. However, additional studies should contemplate other tissues, such as trigeminal ganglia, to provide a more adequate evaluation on the prevalence of such infections.

Virus isolation attempts were unsuccessful in all 18 tonsil samples which bore viral DNA, even after five blind passages in cell culture. However, samples were stored for a long time at 20 °C before being placed at −80 °C. This is known to be highly prejudicial to herpesvirus isolation [43]. However, it could not be avoided on this occasion, since the conditions for storing during sample collection in the Marajó island were far from ideal. Therefore, the data presented here on virus isolation must be interpreted with caution. However, this does not invalidate the findings on the detection of viral genomes. Therefore, the absence of clinical signs with no virus isolation, plus the detection of viral genomes in samples, is quite suggestive that the viruses were present in tonsil tissues in a latent form. Herpesviruses are well known for their capability to establish lifelong latency in various sites, including tonsils [6,7,44]. During the latency, the expression of viral genes is mostly suppressed. As a result, infectious viruses are not produced [45,46]. Nevertheless, during latency, BoAHV1 and BoAHV5 DNA can be detected in the tonsils of latently infected cattle [6,7,44]. In addition, our findings are in agreement with previous work on different alphaherpesviruses, which detected viral DNA in tonsils and other lymphoid tissues of their respective natural hosts, including BuAHV1 [47], canid alphaherpesvirus 1 (CaAHV1) [48], equid alphaherpesvirus 4 (EqAHV4) [49], human alphaherpesvirus 1 (HuAHV1) [50], and SUID alphaherpesvirus 1 (SuAHV1) [51,52]. In each of those studies, the natural hosts were most likely latently infected, since the animals had no clinical signs, and no infectious virus (despite the arguable results at virus isolation) could be recovered from the tissues.

One case of coinfection involving BoAHV1 and BoAHV5 was identified. Co-infections with BoAHV1 and BoAHV5 were reported previously in cattle in Brazil and Argentina [36,53,54]. Moreover, viral recombination events have been demonstrated [53,54]. Thus, in countries where BoAHV1 and BoAHV5 are known to co-circulate, the opportunity for co-infections can be easily foreseen. In fact, reports on recombination events have been focused on viruses recovered from cattle, not bubaline species. Here, we have detected the possibility of co-infections also in water buffaloes. As the three virus types, BoAHV1, BoAHV5, and BuHV1, can infect this species, we speculate that water buffaloes must be included amongst the natural hosts where herpesvirus recombination with the involvement of BoAHV5 might also occur. Unlike buffaloes and cattle, wild animals have not been domesticated, and the cohabitation between wildlife and current animal production systems has been limited. There are practically no data on the occurrence of BoAHV5 in wild animals [41]; therefore, we cannot make assumptions on that. What is apparent is that recombination events do occur between domesticated species. Thus, BoAHV5 may be the result of recombination events that might take place not only in bovine or zebuine, but also in bubaline. Additional interspecies spillover events might then contribute to the spreading of BoAHV5 in bovine, zebuine and bubaline [36]. However, whether such recombinants can be generated in buffaloes or following spillover to bovine and zebuine species, or both, remains to be experimentally determined.

The current study focuses on a particular region of the alphaherpesvirus genome. As documented previously, herpesviruses are prone to recombination events [54,55]. Therefore, analyses based on a single segment of a gene might be prone to errors of interpretation. Unfortunately, the number of full genome sequences of bovine and bubaline herpesviruses is still very scarce. Therefore, for future studies, full genome sequencing must be contemplated in order to evaluate more deeply potential recombination events between these viruses and to allow a more holistic understanding of such interrelations.

Nucleic acid detection methods are usually employed to detect and discriminate the alphaherpesvirus more closely related to BoAHV1. However, these do not allow the differentiation between BoHV-5 and BuHV-1, due to the high degree of similarity of their genomes. The *gC* gene sequences targeted in this study are very dissimilar between BoAHV1, BoAHV5, and BuAHV1, but well conserved within each of these three viral species. For that reason, such a region is suitable for phylogenetic analysis, allowing insights into the viruses’ evolutionary status [56,57,58,59]. The fragment of the gC coding region targeted here was adequate to perform phylogenetic analyses and to allow distinction between the three viruses under study. The nPCR employed here was originally designed to differentiate BoAHV1 from BoAHV5 [56], and has been extensively used in previous studies by our group and by others [36,42,56,60]. The partial region of the gC gene sequenced here corresponds to 37.13% of the complete *gC* gene in BoAHV1 (567 nt), 38.60% in BoAHV5 (564 nt), and 39% in BuAHV1 (564 nt). This methodology allows amplification of a 570 bp fragment, which is followed by an nPCR to discriminate BoAHV1 and BoAHV5. The fragment of BuAHV1 in this same region has a higher identity with BoAHV5, which allows primers to anneal in this region of BuAHV1, leading to amplification. However, sequencing of the amplified fragments was necessary to differentiate between the three viruses, since the high similarity between BuAHV1 and BoAHV5 was still not enough for such differentiation, which was only possible through sequencing of the fragments and by comparing single or short nucleotide substitutions between the amplicons. Phylogenetic analysis showed that the BoAHV1 sequences obtained in this investigation clustered together with the BoAHV1.1 reference strain (NC_063268). Most of the BoAHV5-positive sequences were clustered along with the South American strains of Argentinian and Brazilian origin, except for one sequence that clustered with the Australian BoAHV5 strain (DQ173726).

Moreover, the *UL44* gene segments of BuAHV1 recovered in this study showed high levels of identity between themselves and with the Indian BuAHV1 sequences, while the Australian BuAHV1 and the Italian BuAHV1 clustered in a separate branch. Interestingly, isolates reported in an Argentinian study showed a different profile from our Brazilian sequences [31]. Phylogenetic analysis of eight gene fragments (*UL41*, *UL40*, *UL29*, *UL28*, *UL27*, *UL22*, *US6*, and *US8*) from five Argentinian isolates of BuAHV1 showed that two of the isolates clustered together with the Australian BuAHV1 B6 strain, whereas the other three Argentinian genome sequences clustered together with the sequences of a virus recovered in Italy [31]. These data support the hypothesis that BuAHV1 grouping seems related to the geographical origin of this virus, which, in its turn, is probably related to the importation of bubaline from different regions, at different moments in time. Indeed, the first introduction of buffaloes in Brazil was of animals from Asia in 1890, providing a clue to the clustering of the Brazilian sequences with the Indian isolates.

Lastly, phylogenetic analysis showed that all BuHV1 sequences, including the ones reported in this study, plus the six complete genomes deposited at GenBank to date, were more closely related to BoAHV5 than to BoAHV1. These findings are in agreement with previous studies in Argentina, Iran, and Italy, which also suggested the hypothesis of a common ancestor between BoAHV5 and BuAHV1 [31,32,61]. Remarkably, there are undisputed differences in the worldwide geographical distribution of BoAHV1 and BoAHV5: while BoAHV1 is widely spread in herds around the world, BoHV-5 seems to be concentrated in specific regions, not only in South America, where buffaloes have been introduced in the past two centuries, but also in other regions of the world where buffaloes were introduced. It is proposed here that the most recent common ancestor of BoAHV5 most likely resulted from recombination events between BuAHV1 and BoAHV1 viruses. As no BoAHV5 had been identified previously in buffaloes, common sense previous to this work suggested that recombination would most likely take place in hosts such as bovine, zebuine, or buffaloes. However, the identification of BoAHV5 in bubaline species, as reported here, raises the possibility that BoAHV5 may be generated and eventually be spilled over to bovine, zebuine, or buffaloes. The hypothesis that the BoAHV5 species may have originated following a series of recombination events between BuAHV1 and BoAHV1 is consistent with the geographical distribution of BoAHV5. As no clinically diseased buffaloes were examined here, it is not possible to associate BoAHV5 with disease in that species; thus, it remains to be determined whether this virus type could cause disease in buffaloes.

## 5. Conclusions

In the present study, BoAHV1, BuAHV1, and BoAHV5 infections, including one case of coinfection with BoAHV1 and BoAHV5, were identified in palatine tonsil tissues of clinically healthy water buffaloes. This provides evidence confirming that water buffaloes can not only be infected by these viruses, but, as revealed by the identification of viral genomes and negative virus isolation attempts, the genome-carrying animals were most likely latently infected. As such, these findings suggest that subclinically infected water buffaloes can act as reservoirs for BoAHV1, BoAHV5, and BuAHV1, with the potential to play a role in the generation of recombinants between such viruses. Whether such recombinants can be generated in buffaloes or following spillover to bovine and zebuine species remains to be determined. In countries where co-farming of bovine, zebuine, and bubaline species is increasingly practiced, the proximity of water buffaloes with these other species may at some point in time serve as a source of virus spillover. This must be taken into account to prevent virus dissemination among livestock. Furthermore, for a better understanding of the evolutionary relationships between these viruses (BoAHV1, BoAHV5, and BuAHV1), further studies involving the sequencing of complete genomes are underway.

## Data Availability

The sequences in this study have been deposited in NCBI GenBank under the accession number OR514856-OQ514873.

## Supplementary Materials

The following supporting information can be downloaded at: https://www.mdpi.com/article/10.3390/v16071024/s1, Table S1: Isolate name, country and GenBank accession number of sequences used in this study; Table S2: Percentage of identity of the sequenced segment of the UL44 (gC) gene region between the 18 alphaherpesviruses (BoAHV1, BoAHV5, BuAHV1) reported in this study and equivalent regions of reference herpesvirus strains deposited at GenBank.
