# Supplementary material for: Bovine Alphaherpesvirus 1, Bovine Alphaherpesvirus 5 and Bubaline Alphaherpesvirus 1 in Palatine Tonsils from Water Buffaloes in Northern Brazil and Possible Links with the Origin of Bovine Alphaherpesvirus Type 5
Source: Viruses. 2024 Jun 26;16(7):1024. doi: 10.3390/v16071024 (PMC11281340; doi:10.3390/v16071024)
Supplement: Supplementary file 1 [file viruses-16-01024-s001.zip › viruses-3036326-supplementary.pdf]

Table S1. GenBank accession numbers for the gC genome segments of BoAHV1, BoAHV5, and BuAHV1 recovered in the current study.

Sample Identification	Virus type	GenBank accession number
TO123b	BoAHV1	OR514871
TO122	BoAHV1	OR514872
TO114	BoAHV1	OR514873
TO374	BoAHV5	OR514862
TO338	BoAHV5	OR514863
TO 291	BoAHV5	OR514864
TO 266	BoAHV5	OR514861
TO 143	BoAHV5	OR514865
TO 267	BoAHV5	OR514860
TO 111	BoAHV5	OR514868
TO 219	BoAHV5	OR514870
TO 104	BoAHV5	OR514869
TO 123a	BoAHV5	OR514866
TO 130	BoAHV5	OR514867
TO 117	BoAHV5	OR514859
TO 245	BuAHV1	OR514856
TO 228	BuAHV1	OR514858
TO 362	BuAHV1	OR514874
TO 137	BuAHV1	OR514857

Table S2. Isolate name, country, and GenBank accession number of sequences used in this study.

Isolate Identification	Virus type	GenBank accession number	Isolation Country
C42	BoAHV1.1	MH791336	USA
C47	BoAHV1.1	MH791341	USA
C43	BoAHV1.1	MH791337	USA
C28 55771	BoAHV1.1	MH598938	USA
C35 1839-9847	BoAHV1.1	MH598937	USA
C14 CSU 034-10640	BoAHV1.1	MH598936	USA
LA	BoAHV1.1	MF421714	USA
TSV-2 Nasal MLV vaccine	BoAHV1.1	MH724209	USA
Jura	BoAHV1.1	AJ004801	Switzerland
216 II	BoAHV1.1	KY215944	India
Express 1 IBR MLV vaccine	BoAHV1.1	MH724204	USA
Pyramid IBR MLV vaccine	BoAHV1.1	MH724205	USA
Vista IBR MLV vaccine	BoAHV1.1	MH724206	USA
C29	BoAHV1.1	MH751900	USA
BoviShield IBR MLV vaccine	BoAHV1.1	MH724207	USA
BoviShield Gold FP5 MLV vaccine	BoAHV1.1	MH724208	USA
C18	BoAHV1.1	MH751898	USA
C26	BoAHV1.1	MH751899	USA
C33	BoAHV1.1	MH751901	USA
Titanium IBR MLV vaccine	BoAHV1.1	MH724203	USA
C44	BoAHV1.1	MH791338	USA
Cooper	BoAHV1.1	KU198480	USA
C45	BoAHV1.1	MH791339	USA
C46	BoAHV1.1	MH791340	USA
Nasalgen IP MLV vaccine	BoAHV1.1	MH724210	USA
Arsenal IBR MLV vaccine	BoAHV1.1	MH724202	USA
MN6	BoAHV1.1	MG407780	USA
MN8	BoAHV1.1	MG407782	USA
PA1	BoAHV1.1	MG407790	USA
MN7	BoAHV1.1	MG407781	USA
MN12	BoAHV1.1	MG407786	USA
PA2	BoAHV1.1	MG407791	USA
PA3	BoAHV1.1	MG407792	USA
MN14	BoAHV1.1	MG407788	USA
MN15	BoAHV1.1	MG407789	USA
MN13	BoAHV1.1	MG407787	USA

MN11	BoAHV1.1	MG407785	USA
NVSL	BoAHV1.1	JX898220	USA
NVSL	BoAHV1.1	NC_063268	USA
MN4	BoAHV1.1	MG407778	USA
MN1	BoAHV1.1	MG407775	USA
MN9	BoAHV1.1	MG407783	USA
MN10	BoAHV1.1	MG407784	USA
MN5	BoAHV1.1	MG407779	USA
MN3	BoAHV1.1	MG407777	USA
MN2	BoAHV1.2	MG407776	USA
SM023	BoAHV1.2	KM258882	USA
SP1777	BoAHV1.2	KM258883	USA
K22	BoAHV1.2	KM258880	USA
BHV SHJS	BoAHV1.2	OP035381	China
B589	BoAHV1.2	KM258881	Australia
A663	BoAHV5b	MW829288	Argentina
166/84	BoAHV5b	MZ3642295	Argentina
674/10	BoAHV5b	MZ420492	Argentina
SV507/99	BoAHV5a	NC_005261	Brazil
P160/96	BoAHV5c	KY559403	Brazil
ISO97/45	BoAHV5c	KY549446	Brazil
S101	BuAHV1	OQ669139	India
S102	BuAHV1	OQ669137	India
S103	BuAHV1	OQ608621	India
S104	BuAHV1	OQ669138	India
IZSM	BuAHV1	OQ442798	Italia
B6	BuAHV1	NC_043054	Australia
PRV	SuAHV1	NC_006151	USA

Table S3. The percentage of identity of the sequenced segment of the UL44 (gC) gene region between the 18 alphaherpesviruses (BoAHV1, BoAHV5, BuAHV1) reported in this study and equivalent regions of reference herpesvirus strains deposited at GenBank.

**Isolate Id***	**Reference Strains**					
	**BoAHV1.1 (NC_063268)**	**BoAHV1.2 (KM258881)**	**BoAHV5 (NC_005261)**	**BuAHV1** (NC_043054)	**BuAHV1** (OQ669139**)	**SuAHV1** (NC_006151)
**TO123b**	**99,6%**	**99,2%**	**92,2%**	91,8%	91,7%	57,1%
TO122	99,6%	99,2%	92,2%	91,8%	91,7%	57,1%
TO114	99,1%	98,7%	92,7%	91,8%	92,2%	57%
TO374	92,5%	92,5%	98,9%	95,3%	95,9%	56,8%
TO338	92,5%	92,5%	98,9%	95,3%	95,9%	56,8%
TO 291	92,5%	92,5%	98,9%	95,3%	95,9%	56,8%
TO 266	92,4%	92,4%	98,7%	95,2%	95,7%	56,8%
TO 143	92,4%	92,4%	98,7%	95,2%	95,75	56,6%
TO 267	92,7%	92,7%	98,7%	95,5%	96%	57%
TO 111	92,9%	92,9%	99,2%	95,7%	96,2%	57,1%
TO 219	92,9%	92,9%	99,2%	96%	96,2%	57,1%
TO 104	92,9%	92,9%	99,2%	95,7%	96,2%	57,1%
TO 123a	93,2%	93,3%	98,9%	92,4%	96,2%	57,3%
TO 130	93,2%	93,2%	98,9%	96%	96,6%	57,3%
TO 117	91,3%	91,3%	96,8%	97,5%	98,5%	58,5%
TO 245	91,7%	92%	95,7%	98,5%	100%	59,6%
TO 228	91,7%	92%	95,7%	98,5%	100%	59,6%
TO 362	91,3%	91,7%	95,3%	98,2%	99,6%	59,3%
TO 137	91,7%	92%	95,7%	98,5%	100%	59,6%

* Sequences obtained in the present study. ** Indian strain (S102).
